# Electrochemical coating of dental implants with anodic porous titania for enhanced osteointegration

**DOI:** 10.3762/bjnano.6.224

**Published:** 2015-11-20

**Authors:** Amirreza Shayganpour, Alberto Rebaudi, Pierpaolo Cortella, Alberto Diaspro, Marco Salerno

**Affiliations:** 1 Nanophysics Department, Istituto Italiano di Tecnologia, via Morego 30, 16149 Genova, Italy; 2 Rebaudi Dental Office, piazza della Vittoria 8, 16121 Genova, Italy,; 3 Odontosalute, via Edmondo de Amicis 2, 16122 Genova, Italy

**Keywords:** anodization, dental implants, nanopores, surface treatment, titania

## Abstract

Clinical long-term osteointegration of titanium-based biomedical devices is the main goal for both dental and orthopedical implants. Both the surface morphology and the possible functionalization of the implant surface are important points. In the last decade, following the success of nanostructured anodic porous alumina, anodic porous titania has also attracted the interest of academic researchers. This material, investigated mainly for its photocatalytic properties and for applications in solar cells, is usually obtained from the anodization of ultrapure titanium. We anodized dental implants made of commercial grade titanium under different experimental conditions and characterized the resulting surface morphology with scanning electron microscopy equipped with an energy dispersive spectrometer. The appearance of nanopores on these implants confirm that anodic porous titania can be obtained not only on ultrapure and flat titanium but also as a conformal coating on curved surfaces of real objects made of industrial titanium alloys. Raman spectroscopy showed that the titania phase obtained is anatase. Furthermore, it was demonstrated that by carrying out the anodization in the presence of electrolyte additives such as magnesium, these can be incorporated into the porous coating. The proposed method for the surface nanostructuring of biomedical implants should allow for integration of conventional microscale treatments such as sandblasting with additive nanoscale patterning. Additional advantages are provided by this material when considering the possible loading of bioactive drugs in the porous cavities.

## Introduction

Titanium (Ti) is the standard material used for dental and orthopedic implants, thanks to its very good strength, corrosion resistance and biocompatibility [1–2]. Despite the very high success rate of Ti dental implants (>90%), there is still room for optimization of osteointegration, particularly for diabetics, smokers and oncology patients [3]. As with most metals, Ti in wet or even ambient air environment develops a thin layer of native oxide, namely TiO_2_ (titania). While it is generally recognized that surface topography is a major factor for osteointegration of all implants [4], the lower surface energy of titania as compared to that of alumina and silica for example [5], makes micro-nanoscale patterning of this material of even more critical importance for implant success.

The surface micropatterning of Ti implants is usually achieved by mechanical (sandblasting) or purely chemical (etching) treatments [4]. However, Ti is also known as a valve metal, similar to the more common Al [6]. As such, electrochemical (EC) anodization of Ti, which is a combination of metal etching and oxide growth in the presence of applied voltage, results in the formation of porous titania nanotubes grown perpendicular to the metal – a material called anodic porous titania (APT).

APT is usually of interest for applications in catalysis or optoelectronics [7]. Here we present its use as a coating for nanopatterning the surfaces of dental implants. One advantage of APT for applications in biomedicine is with respect to its analogue obtained on Al, namely, anodic porous alumina (APA), which is mainly used in nanotechnology [8] because no particular pore order is required in this field. Here, a relatively uniform pore size and spacing is required and thus the preliminary electropolishing and two-step anodization used for APA to form hexagonal pore arrays are not necessary [9]. A single anodization is sufficient and from this perspective, may represent a simple and inexpensive nanopatterning procedure for biomedical Ti.

In fact, the application of nanoporous oxides as biological surfaces (where living cells should adhere and grow) has been already explored for APA also [10–12]. Generally speaking, oxide inertness provides biocompatibility, while controlled porous patterning allows for tuning the roughness for optimized stimulation of living-cell response. The role of nanotopography in guiding cell differentiation and tissue generation is not fully understood yet, but is a well-known phenomenon [13–15]. In extreme synthesis, a nanorough substrate with possible adhesion/growth factors mimics the extracellular matrix [16].

Since Ti is used for most permanent implants, interest in nanopatterning biomedical surfaces with anodization has recently shifted from Al (i.e., APA) to Ti (i.e., APT [17–19]). However, the anodization of Ti implants poses several challenges: the Ti used for implants is not ultrapure (as is used in basic research), but is rather an alloy, and the medical implants are not flat surfaces, but are 3D objects with curved surfaces. Therefore, even though positive results have been recently achieved [20–22], the transfer of the required processes from laboratory specimens to real implants is not trivial.

The fabrication of APT in itself forms an organized film where the pores grow in a columnar form with the oxide according to mutual interaction in a form of self-assembly. In addition, further opportunities for surface organization are provided by subsequent functionalization of the APT with functional overcoating layers of bioactive materials, eventually using the pores as a template. Here we report on APT fabrication for dental implants and give an example of pore loading with a bioactive element.

## Results and Discussion

**Figure 1:**
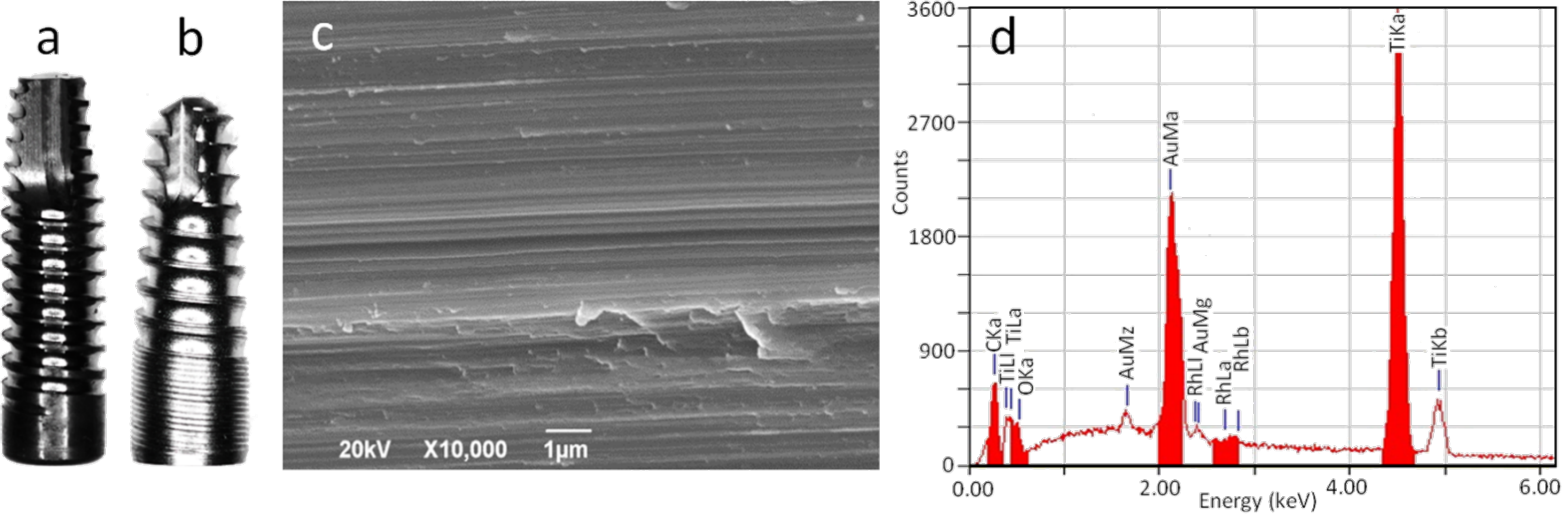
(a,b) Pictures of the implants used in this experiment, (a) Premium Straight from S&M and (b) Stark. (c) Typical SEM micrograph of the surface of the implants, as received. (d) Corresponding EDS spectrum of an S&M implant, as received.

In Figure 1a,b pictures of the two types of implants investigated, Sweden & Martina (S&M, Figure 1a) and Stark (Figure 1b), are shown. They are both conical implants of similar geometry and same nominal Ti purity, namely grade 4 and both available in machined-only form (i.e., not finished with the final roughening treatment). Indeed, under scanning electron microscope (SEM) imaging at high (10,000×) magnification they looked the same with the typical lay of machined pieces (Figure 1c). Concurrently, the chemical composition, as assessed by energy dispersive spectroscopy (EDS), also looked quite similar. In Figure 1d the EDS spectrum of a typical S&M implant is shown. It appears that, in addition to Ti from the bulk implant core and gold (Au) from the coating deposited for SEM analysis, traces of additional chemical elements are present, such as carbon, ascribed to organic contamination during packaging, and, to a lower extent, oxygen, ascribed to native metal oxide on the surface. Contamination of rhodium also appears at very low levels (estimated ≈0.8 at %), which was the same for both types of implants. According to the producing companies, these contaminates may be associated with the machining tools and/or the final washing, or possibly due to cross-contamination among different manufacturing processes carried out with the same equipment. In the case of Stark implants, a minor difference in the spectrum is the occurrence of another minor peak assigned to Al (see Supporting Information File 1, Figure S1). While both Ti materials should be of commercial grade 4 (and not grade 5, also called Ti6Al4V, which contains 6 wt % Al), our interpretation is that the detected Al is yet another contaminant appearing during the bulk metal machining. Al cannot even be associated with sandblasting by means of alumina abrasive particles, since it appears on just-machined implants.

**Figure 2:**
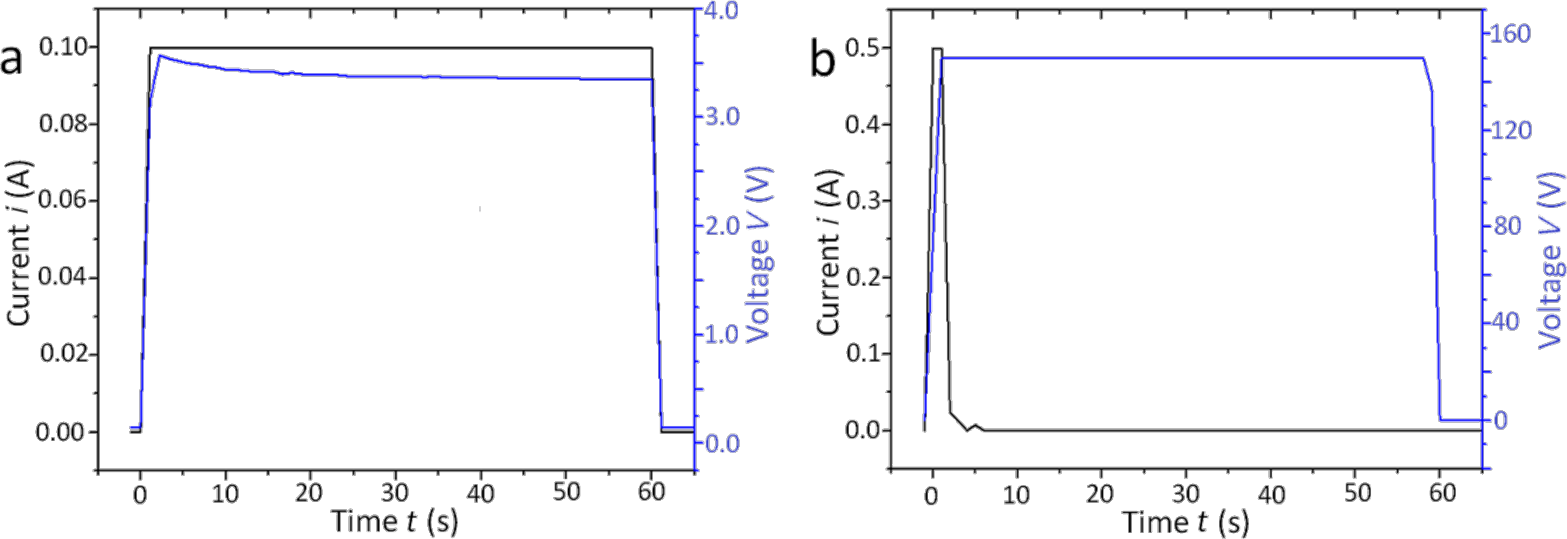
Plots of both current and voltage during the surface modification of the dental implants. (a) Preliminary, optional cathodization pretreatment, and (b) standard, subsequent anodization. The current, *i*, is plotted in black, the voltage, *V*, in blue. The curves were obtained from the anodization of Stark implants, but were very similar for S&M implants as well.

Both types of implants were subjected to two slightly different processes, both carried out at room temperature (RT) for 1 min: in one case only anodization was applied (Ti positive, voltage +150 V), while in the other case, this step was preceded by inverted polarization (voltage −150 V). The latter step, called cathodization, should protonate the surface and make anodization more effective according to some literature [23]. The typical chronopotentiometric and chronoamperometric profiles of these processes are shown in Figure 2a,b for the cathodization pretreatment (where applied) and the standard subsequent anodization, respectively.

It should be mentioned that the electrochemical settings involved both a target voltage, *V*_t_, and a target current, *i*_t_. When the power supply is switched on, both quantities are simultaneously raised and the one that first reaches the target value is clamped. Therefore, also partly due to the peculiar shape of the anodized element, it is not always possible to define the whole process as either potentiostatic or galvanostatic. During this process the conditions may change, making one target value easier to attain than the other at a given electrolyte conductivity and temperature.

Within intermediate ranges of the above quantities (e.g., 20–180 V and 0.1–1.5 A), thus avoiding the burning regime, the selected temperature is only of secondary importance with respect to the applied voltage (or current). Nevertheless, for proper interpretation of the results, it is important that the temperature be kept as constant as possible, regardless of its value. For this reason we chose a temperature close to typical RT.

From Figure 2a it appears that the cathodization pretreatment lasted for the whole duration period under conditions of constant current (i.e., a galvanostatic process) and almost constant voltage as well, and this was the same for both implant types. In particular, despite the selected *V*_t_ = 150 V applied to the cathode, a value of only ≈3.5 V was reached, given the low *i*_t_ = 0.1 A set as a protection limit against unwanted side effects.

The profiles for anodization were similarly equivalent for the different implants, yet different from the cathodization, as shown in Figure 2b. In all cases, anodization was self-terminated after a very short time of 2–4 s. When the total passed charge was calculated, it appeared to be always comparable (≈12 C), which sets a limit to the surface coating of the implants with the passivating anodic oxide of ≈0.1 C/mm^2^.

The fact that a longer anodization time does not increase the APT thickness is different from what is known for APA [24], where the oxide continues to grow at the expense of the underlying metal. In fact, in previous extensive studies, the nature of the porosity resulting in Ti from anodization was also described to be of a different nature than for Al [25]. The APT pattern was ascribed to a pitting regime of anodization, occurring above the electrical breakdown threshold for the material, which would probably account for the more disordered appearance of the emerging pores with respect to those of APA.

**Figure 3:**
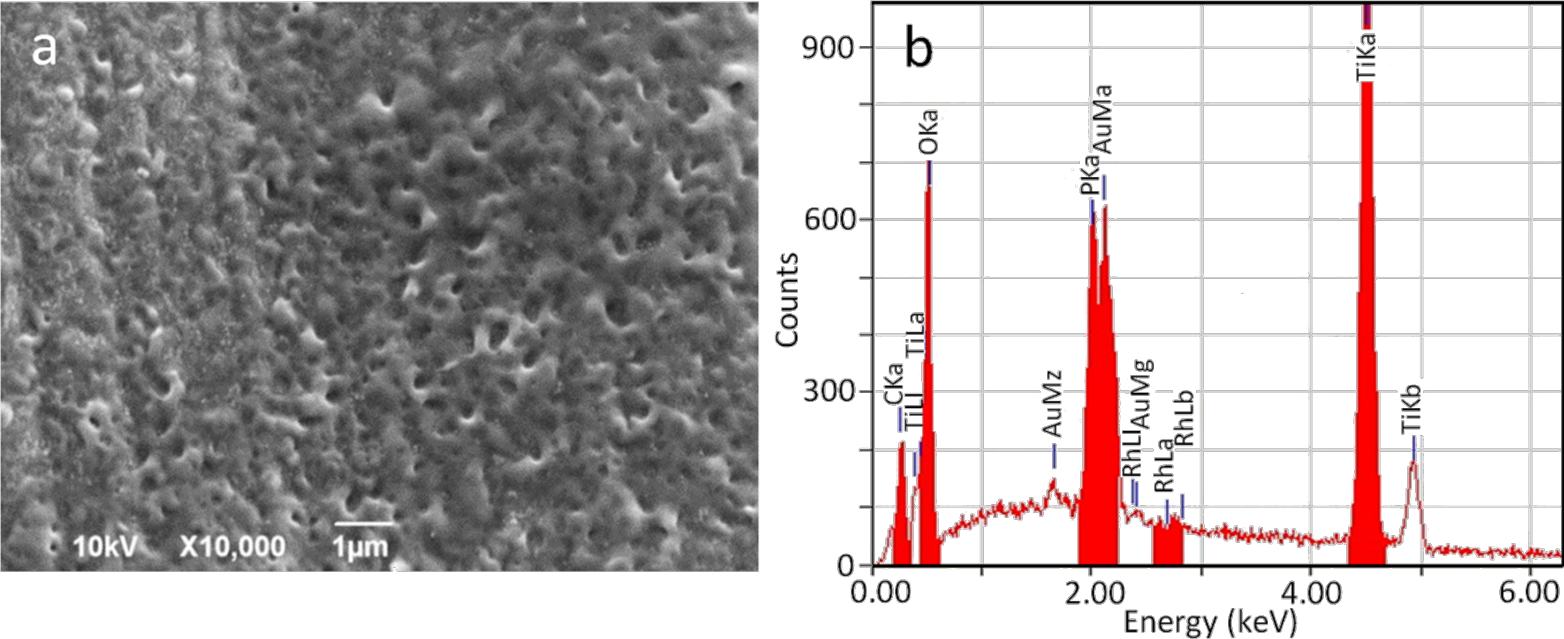
Typical results of anodization for both implant types, with or without cathodization pretreatment. (a) SEM micrograph of the surface showing the characteristic APT pores and (b) EDS spectrum, showing the increased O content and P contamination.

In Figure 3 the typical results of anodization are shown with respect to both the surface morphology (SEM, Figure 3a) and its composition (EDS, Figure 3b). The reported data are from S&M implants, but equivalent results were obtained for Stark implants. In Figure 3a, the characteristic nanoporosity of APT appears. The pores, which are rather irregular, present a broad size distribution, with a diameter of 100–200 nm. The underlying lay of machining is still visible, although partly obscured by the nanopatterning. Similar pores were obtained in our group on ultrapure Ti (see Supporting Information File 1, Figure S2). In that case, the temperature was lower and sulphuric acid was used instead. As a result, the pore edges were sharper, while here we have rounded pore mouths, in agreement with literature results [26].

The comparatively large pore size, at the border between nano- and micro-scale, is associated with the high anodization voltage used here, which should be proportional to the pore size, similar to the case of APA [7]. This high voltage has been selected because, according to Choi [25], no pore formation occurs at a voltage below ≈100 V, even if formation of oxide is still observed. At the same time, it has also been observed in in-vitro experiments that too small nanopores can even be detrimental to living cell adhesion, as they may give rise to a kind of hydrophobic and thus antiwetting behavior [27].

In Figure 3b, the EDS analysis confirms the presence of Ti oxide on the surface. In fact, the O content significantly increased with respect to Figure 1d from ≈3 at % up to ≈14 at %. This is consistent with the presence of surface titania with a thickness significantly higher than native oxide. A stoichiometric ratio to Ti cannot be obtained from EDS (in fact Ti is still dominant, ≈73 at %), given the deep penetration (≥1 µm) of the energetic, primary electrons (10 kV) with respect to the APT thickness (≈100 nm), such that the probed volume is mainly in the bulk of the Ti implant under the surface.

In Figure 3b a new type of contamination, phosphorus, also appears at a concentration as high as ≈10 at %. In fact, from APA fabrication, it is also known that some amount of electrolyte anions (typically 3–8 wt %) are incorporated into the porous oxide during anodization [6]. The same applies also for the anodization of Ti, and thus the observed P has to be ascribed to phosphate anions PO_4_^2−^ entrapped within the porous oxide during its growth. Actually, this is the reason why we decided to use phosphoric acid as the anodizing electrolyte, since phosphate is likely to be biocompatible and even bioactive in the foreseen application of the coatings for osteointegration, due to its affinity to tri-calcium phosphates or hydroxylapatite.

Both the Stark and S&M implants were made using grade 4 Ti. Actually, grade 5 Ti is commonly used only for abutments and other parts, since it presents higher mechanical performance (e.g., yield stress of 860 MPa vs 550 MPa) but is less biocompatible [1].

**Figure 4:**
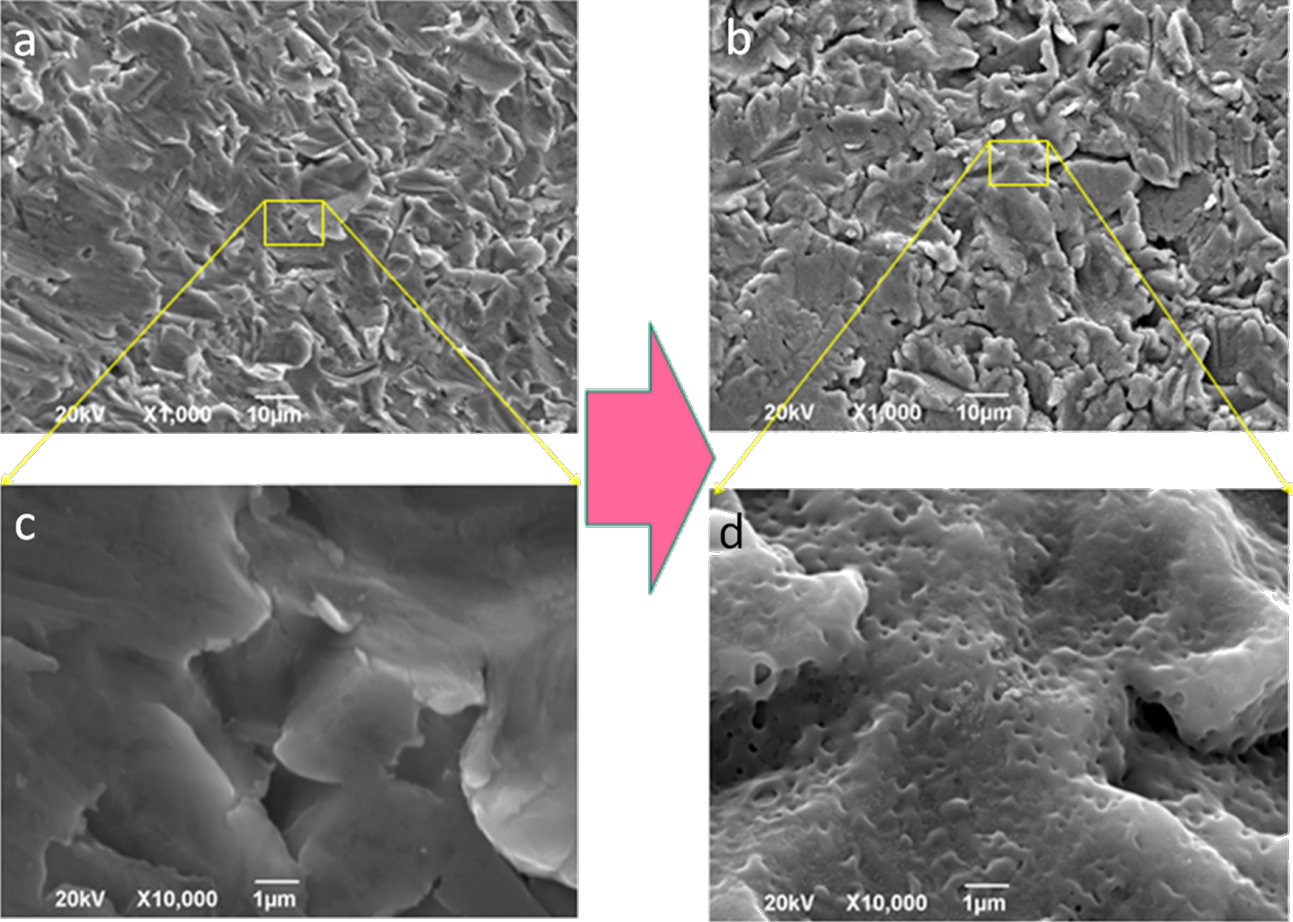
SEM micrographs of finished Stark implants, before (a,c) and after (b,d) anodization, at 1,000× (a,b) and 10,000× (c,d) magnification. The high magnification images are close-up views extracted from the corresponding regions (see yellow lines) of the same area. The large arrow represents the anodization process.

The successful patterning of Ti by means of oxide nanopores, as shown in Figure 3a on machined implants, can be of greater importance when demonstrated on implants patterned on the microscale by the different and most common methods of either sandblasting and/or acid etching (simple wet etching in the absence of driving electrical field). In fact, it is possible that a combination of both roughening scales, the micro- and the nano-, may be the most effective procedure for osteointegration of the pristine Ti surface. Therefore, in a separate set of experiments, we also anodized implants of both types (S&M and Stark) that were already patterned according to the standard technique of the respective company. Anodization was confirmed to be successful in formation of nanopores on the micropatterned Ti implants also. In Figure 4 we report the case of Stark implants, but similar results were also obtained for the S&M implants.

Figure 4 shows the SEM micrographs of finished Stark implants after their standard process of sandblasting. We can see similar areas before (left) and after (right) anodization. Additionally, in both cases, the same area pictured at low magnification (top) has been taken at higher magnification (bottom). Interestingly, at lower magnification the same microscale roughness is observed before and after anodization (Figure 4a,b), meaning that the treatment did not destroy it. At the same time, at high magnification, the typical nanopores due to APT appear on the anodized implant (Figure 4d) and were not present before anodization (Figure 4c). In Figure 4d these nanopores are obviously overlaid onto the microscale roughness due to sandblasting. The effectiveness of the anodization treatment is thus confirmed, which is important in view of real manufacturing carried out in combination with the standard microscale patterning processes of sandblasting and wet etching.

According to both SEM morphology and EDS composition, no significant difference was observed on average after inspection of several different locations on different implants (N ≥ 6), and between implants pretreated or not pretreated with cathodization. This pretreatment should result in loading the Ti with hydrogen, which would decrease the oxide breakdown voltage, and thus increase the pore density at constant potential. However, we observed no significant change in pore size with the pretreatment. The previous authors who used that [23] applied the same anodic potential as set here but a different electrolyte, namely 1 M sulphuric acid. However, the major difference, and the possible reason for the lack of this effect in our case, could be the low current limit of 0.1 A set here.

**Figure 5:**
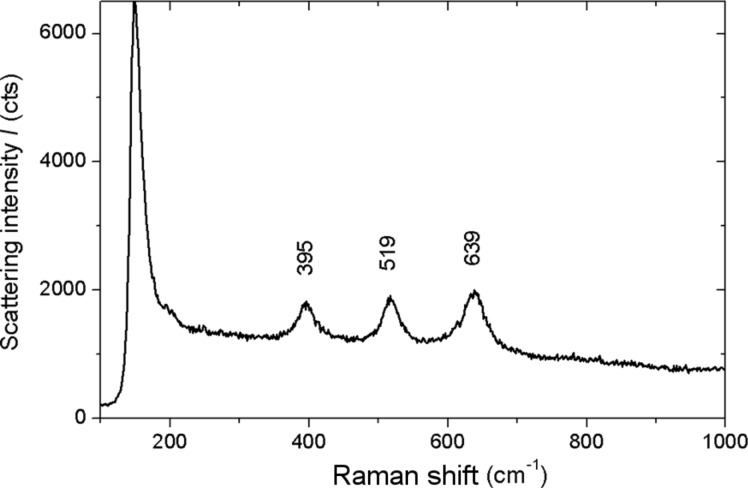
Typical uncorrected Raman scattering spectrum of an anodized implant, representative of all implant types with or without pretreatment.

In any case, the pretreatment should not change either the crystalline phase of the formed APT or its thickness. With or without pretreatment, Tanaka et al. [23] observed a combination of anatase and rutile for APT by means of X-ray diffraction spectroscopy. On the other hand, Choi states in his extensive work that rutile is formed at an anodization voltage as high as 150 V, while amorphous titania is obtained at lower voltages [25]. The crystalline phase of APT is of some importance since it seems that, with respect to osteointegration, anatase is preferred over rutile [28]. Unfortunately, if rutile is formed upon anodization, no existing easy route is known to convert this more stable form of titania back to transient anatase. We performed Raman spectroscopy on both types of anodized implants, those with or without pretreatment. Again, we observed no major differences in the different cases. For all measurements, given the limited thickness of APT, we had to use collecting conditions of low magnification (objective of 10×), high laser power (≈100 mW) and long accumulation time (1 min) to obtain spectra with reasonable signal to noise ratio. A representative Raman spectrum is presented in Figure 5.

According to the triplet peaks appearing in Figure 5 at around 395, 519 and 639 cm^−1^ and upon comparison with former data available in the literature for Raman analysis of anatase titania [29–30], the APT formed in our case seems not to be rutile but rather anatase. This is in agreement with the results of anodization of Ti by a different group [31].

The spontaneous phosphate incorporation occurring during anodization in H_3_PO_4_ is not the only form of doping that may be exploited in order to increase the possible bioactivity of the APT coating. In fact, one may explore the possibility to add different chemical species to the APT coating from those naturally resulting in the dissolved acid electrolyte. Interesting candidate elements are F, Ca and Mg and P, as demonstrated by recent loading carried out on implants coated with nanoporous titania by means of different techniques [22,32–33]. We selected Mg, which is essential to all living cells for its interaction with polyphosphate compounds such as ATP, DNA and RNA, required by many enzymes for their functioning, and present in many pharmaceutical products. In the research literature, Mg has also been added to hydroxylapatite to support to bone formation but with varying results (e.g., positive in [34] and missing in [35]).

**Figure 6:**
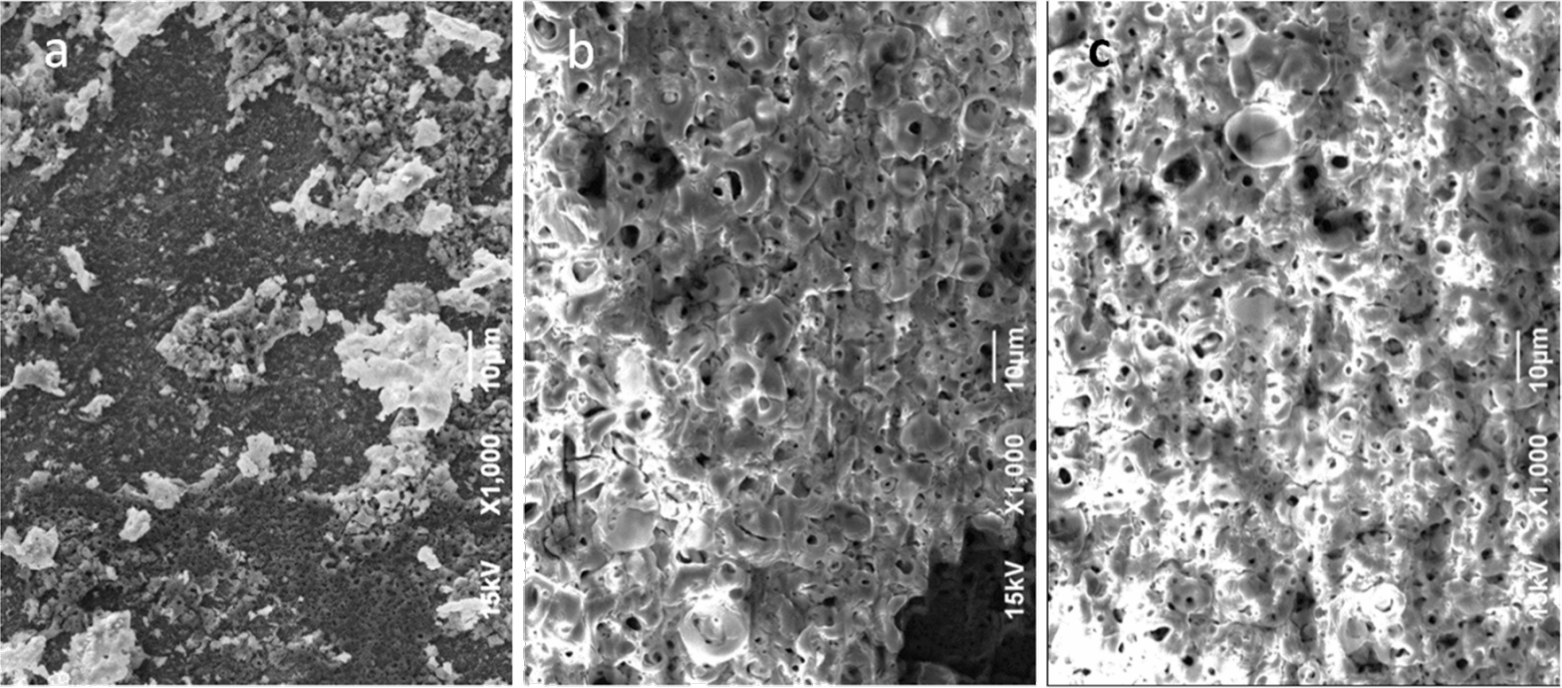
SEM micrographs of implants (S&M) anodized in the presence of a Mg additive. (a) 0.5 M Mg, 1 min anodization; (b) 1.5 M Mg, 1 min anodization; (c) 1.5 M Mg, 10 min anodization.

With the goal of the incorporation of Mg in the APT coating, in a separate set of experiments, we added magnesium sulfate (MgSO_4_) to the electrolyte. The salt was added in two different concentrations (0.5 and 1.5 M) and for two different processing times of the subsequent anodization (1 and 10 min). The presence of Mg was confirmed by means of SEM and EDS. In Figure 6 representative SEM micrographs of the implant surfaces after anodization with the Mg additive at an intermediate magnification (1,000×) are shown.

In Figure 6a, it appears that the lower Mg concentration (0.5 M) for the short anodization time (1 min) gave rise to surface aggregates covering only a minor portion of the surface, (20–30%, the image is representative). In Figure 6b, the effect of increasing the concentration to 1.5 M while keeping the anodization time at 1 min appears to result in an almost full coverage of an aggregated overlayer (80–90%). Concurrently, despite the standard overcoating with 10 nm Au (in order to avoid the static charging effect due to the electron beam), the enhanced contrast still appears to be an effect of the thick insulating coating on top of the anodized Ti. As shown in Figure 6c, the case of high concentration (1.5 M) and long anodization time (10 min) further increases the overlayer coverage, reaching ≈100% in all regions (N ≥ 3).

EDS also confirmed the overlayer aggregates comprised of Mg. A quantitative EDS analysis pointed out that in the above three cases of Figure 6, the Mg content detected was ≈0.5, ≈1.0 and ≈1.3 at %, respectively. The entire amount of Mg found may not necessarily be incorporated in the APT or loaded in the APT pores. However, anodizing seems to enhance this functionalization. Indeed, when control implants were submerged after anodization in Mg solutions with same concentration and for the same soaking time, we observed a lower Mg content of ≈0.1, ≈0.3 and ≈0.6 at %, respectively. The reason why Mg is more effectively coated on the APT when added during anodization is not clear, since this is a cation, and in principle, should be preferentially driven to the cathode. However, we may suppose that at least electrically neutral species containing Mg are also formed in the electrolyte, such as Mg_3_(PO_4_)_2_ or, more likely, Mg(OH)_2_. These species with comparatively large size and low mobility could be trapped within the flow of the other anions.

**Figure 7:**
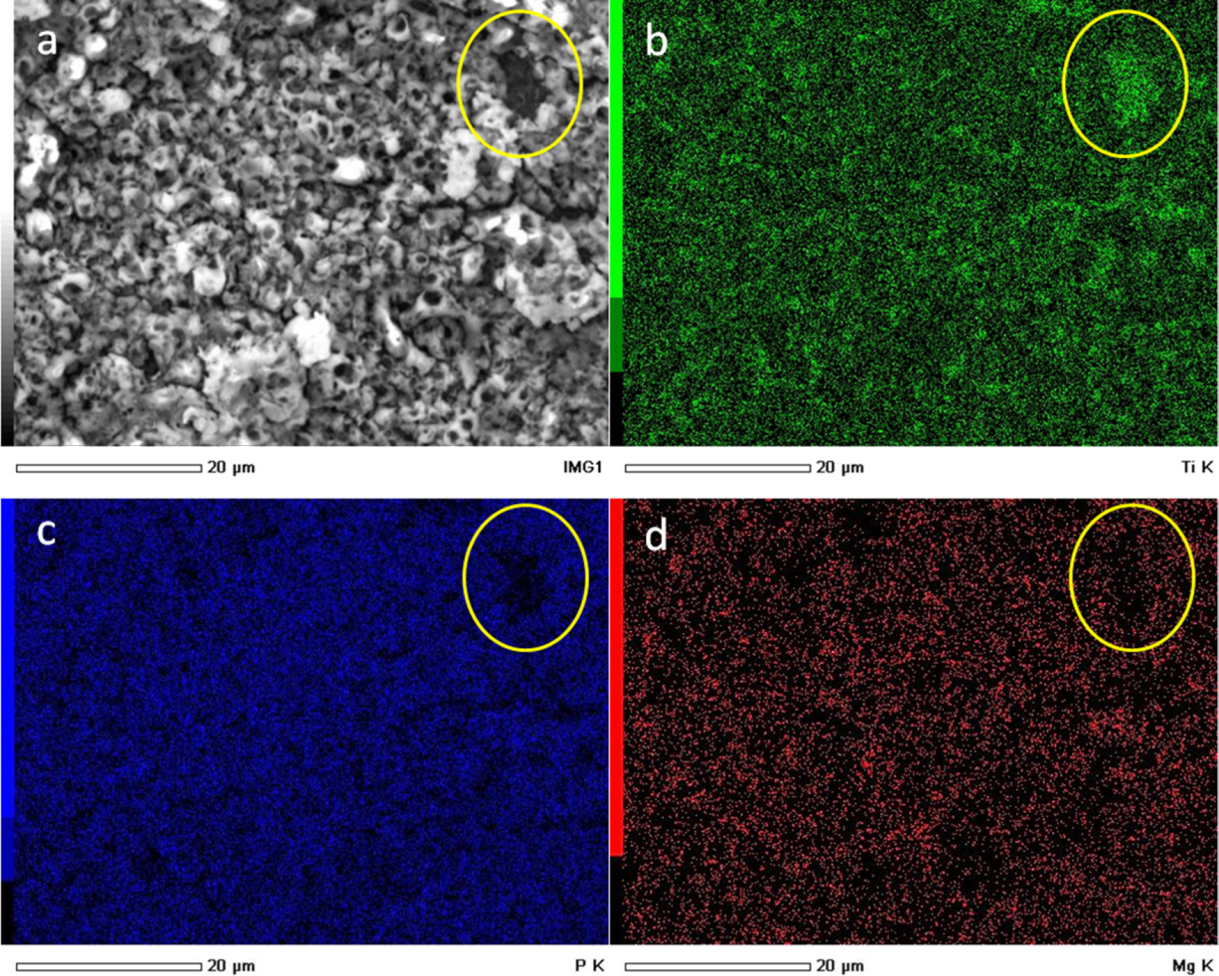
SEM and EDS mapping of the elemental species on the surface of implants (S&M) anodized in the presence of 1.5 M Mg for 1 min. (a) SEM (backscattered electron) morphology, (b) Ti map, (c) P map, and (d) Mg map.

An example of EDS chemical mapping is presented in Figure 7. It appears that all the elemental species of interest, namely Ti, P from the electrolyte phosphate and Mg additive, are evenly distributed over the surface (and the same is for O, not shown).

Occasional defects appear, such as in the top right region of Figure 7 (circled in yellow). The depressed (dark) region on the morphological image (Figure 7a) probably corresponds to an area of missing or thinner Mg coating. Indeed, the Mg map (Figure 7d) shows a locally lower level in that spot. The Ti (Figure 7b) is apparently more concentrated there, due to the decreased screening effect of the Mg overlayer (a similar effect was observed for the map of O). However, the P content (Figure 7c) is also lower in that spot, meaning that not only the Mg was coated less efficiently but also the APT did not form in that site. This can be ascribed to the presence of different contaminants on the Ti (e.g., C, not mapped here). Therefore, where no APT forms, the decreased ionic flow lines also prevent formation of a good Mg overlayer. However, overall, a high coverage of Mg is obtained on the surface.

As shown in Figure 6a, the Mg overlayer results in apparent clogging of the APT pores. This may cause the implant to lose the desired nanostructure patterned by the anodization, at the cost of gaining the chemical functionalization of Mg. However, at a longer anodization time, the Mg overlayer itself seems to form a nanoporous structure. These pores are on average 5–10 times larger than those in the pristine APT (i.e., on the microscale). We make the hypothesis that the coating is porous due to local percolation of the ionic current through it, with the arrangement of such conduction channels favoring the merging into large pores. In any case, the underlying structure of APT nanopores, which is made in an inorganic inert material, is not lost, and will eventually be revealed after bio-utilization of the Mg overlayer at an implant site, providing interaction with the host tissue.

## Conclusion

Anodization in acid electrolyte provides a conformal coating with pores also formed on curved surfaces of commercial grade Ti, and is thus a feasible approach for the nanopatterning of dental implant surfaces.

The implants of the same Ti grade from the two different companies considered here did not present significant differences.

In our experience, the cathodic pretreatment did not provide different morphological, compositional or structural results for the subsequent anodization at the given electrolyte concentration, bath temperature, and applied voltage and current conditions.

The nanoporosity resulting from the anodization did not alter the underlying surface profile on the microscale. Therefore, when not sufficient to provide osteointegration by itself, anodization can be an additive treatment in addition to microscale sandblasting or wet etching.

By anodizing in phosphoric acid, a remarkable doping of the fabricated APT coating with phosphate ions occurs spontaneously, which can be already considered as a biofunctionalization of the nanoporous surface. Further in situ functionalization aiming at improved bioactivity of the nanoporous surfaces may be obtained by using additive species in the anodizing electrolyte, such as Mg as shown here. The amount of functionalization obtained with Mg additive is approximately a factor 10 less effective than for the phosphate anions of the electrolyte base acid, and optimization conditions should be sought for optimal Mg adsorption.

The as fabricated APT was found to be at least partly anatase, which is desirable for osteointegration. A higher crystallinity, with removal of water and residual amorphous APT phase, may likely be obtained by post-fabrication annealing at moderate temperatures to avoid the transition to rutile.

The next step in our research will be the further functionalization of the organized porous film surface with bioactive molecules such as proteins and adhesion factor, and in vitro experiments with osteoblast-like cell cultures.

## Experimental

### Implants used

We used dental implants from two different manufacturers, Sweden & Martina (S&M) and Stark. From the range of available S&M implants, the type Premium Straight was selected. Both implant types were of grade 4 Ti and were provided in machined form only (i.e., without surface treatment for roughening and osteointegration). The implants were sterilized and sealed in plastic boxes, and were used as received, taking care to minimize contamination prior to anodization, by handling only with clean tweezers and gloves. For mounting in the anodization setup, we took advantage of the metallic screw available for connection of the abutment, which fit the inner base cavity of the implants.

### Anodization

The implants were suspended vertically upside-down and submerged in a 1.5 M aqueous solution of phosphoric acid H_3_PO_4_ (Sigma Aldrich, Milan, Italy). The counter electrode (normally the cathode) was an inert Pt wire (1 mm thickness), curled in a spiral to form an almost compact circle of ≈3 cm in diameter in front of the implants at a distance of ≈2 cm. The anodization was carried out by means of a high power supply (N5751A, Agilent Technologies, USA), connected for both control and output to a laptop computer. A Visual Basic macro allowed for the collection of both current (*i*) and voltage (*V*) data from the circuit. The anodization was carried out in prevailing potentiostatic mode, by setting the target voltage and target current to 150 V and 0.5 A, in a double-walled beaker with circulating silicone oil, kept at an approximately constant temperature of 25 °C (RT) by means of a thermocryostat RP 3530 (Lauda, Germany). The cathodization treatment, when applied, was set 150 V and applied to the Pt counter electrode (with the implant set to ground) and 0.1 A target current.

### Imaging and chemical analysis

Both types of implants were characterized before and after anodization by SEM. They were mounted on Al stubs by means of double-sided adhesive carbon tape and overcoated with a ≈10 nm thick layer of sputtered gold before inserting into the SEM chamber. We used a JSM-6490LA (JEOL, Japan) instrument equipped with energy dispersive spectroscopy (EDS) for the chemical identification of the coating composition. For imaging, the instrument was operated at a primary electron beam energy of 20 kV, aperture 2, and spot size 30, while for EDS we used 10 kV, aperture 3 and spot size 60, respectively.

Additionally, on implants not coated with gold, we used a micro-Raman Raman spectrometer (inVia, Renishaw, UK) equipped with the software program WiRE 3.4 to assess the surface resulting from the anodization. A 785 nm wavelength laser was used together with a 1200 grooves/mm grating, collecting spectra with a 10× objective in the 100–3200 cm^−1^ range. The incident power was ≈100 mW with an accumulation time of 1 min.

## Supporting Information

File 1 Additional experimental information.
