# Supplementary material for: Electrochemical coating of dental implants with anodic porous titania for enhanced osteointegration
Source: Beilstein J Nanotechnol. 2015 Nov 20;6:2183–92. doi: 10.3762/bjnano.6.224 (PMC4660911; doi:10.3762/bjnano.6.224)
Supplement: File 1 — Additional experimental information. [file Beilstein_J_Nanotechnol-06-2183-s001.pdf]

Supporting Information

for

Electrochemical coating of dental implants with anodic porous titania for enhanced osteointegration

Amirreza Shayganpour[1], Alberto Rebaudi[2], Pierpaolo Cortella[3], Alberto Diaspro[1], and Marco Salerno[1,*]

[1]Nanophysics Department, Istituto Italiano di Tecnologia, via Morego 30, 16149 Genova, Italy, Rebaudi Dental Office, piazza della Vittoria 8, 16121 Genova, Italy, and [3]Odontosalute, via Edmondo de Amicis 2, 16122 Genova, Italy

Email: Marco Salerno - marco.salerno@iit.it

* Corresponding author

Additional experimental information

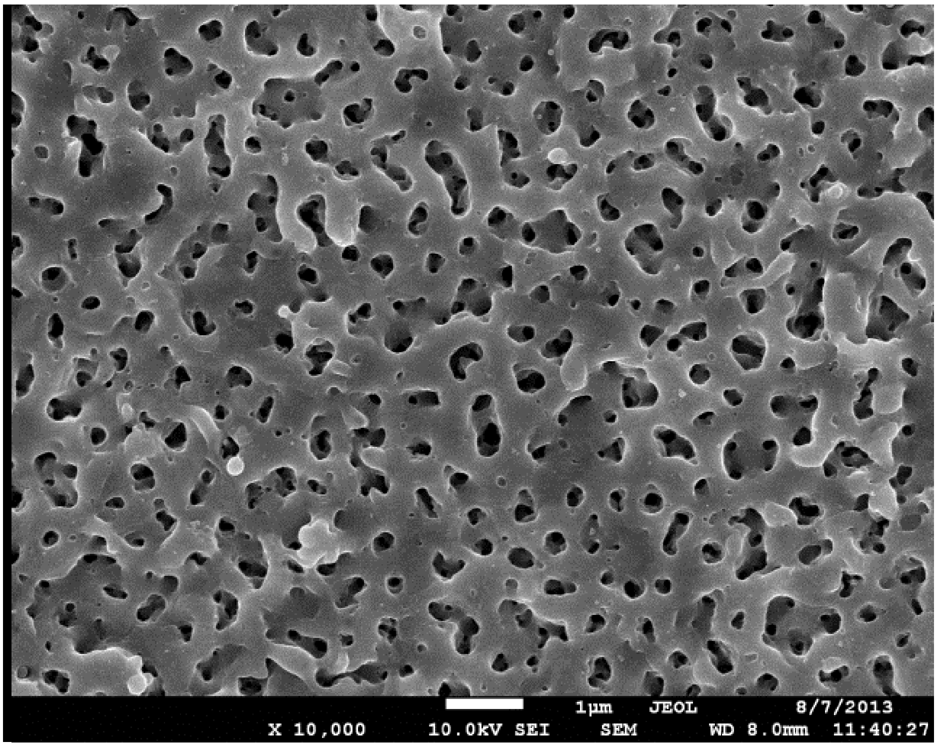

Figure S1: SEM top-view of APT from anodization of thick ultrapure Ti plate, under 150 V, in 1 M sulphuric acid, at 9 °C for 1 min.

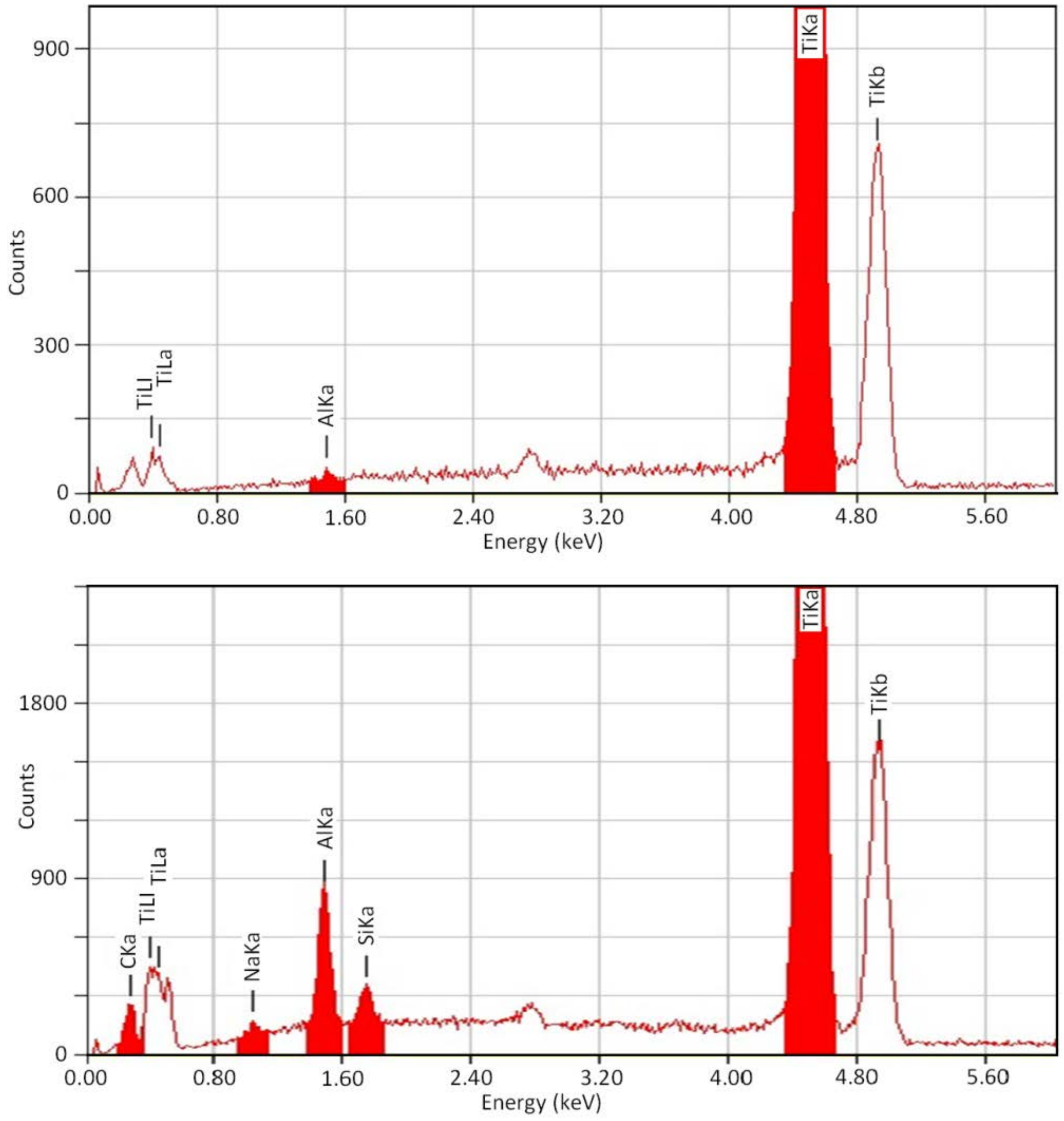

Figure S2: Top: EDS of Stark machined implant. The presence of Al appears despite the Ti being grade 4 and not grade 5. Bottom: EDS of same type of implant as finished (i.e., not only machined). In addition to some Na, which should be there as residual of biodegradable sandblasting salt particles, Si also appears, and the Al contents is significantly increased. This makes one think that also inorganic (alumina and silica) abrasive particles are used.
